# Fundamental features of AlCl_4_^−^-/AlCl_4_-graphite intercalation compounds of aluminum-ion-based battery cathodes

**DOI:** 10.1039/d2ra06079e

**Published:** 2022-12-23

**Authors:** Wei-Bang Li, Shih-Yang Lin, Ming-Fa Lin, Vo Khuong Dien, Kuang-I. Lin

**Affiliations:** Department of Physics, National Cheng Kung University Tainan Taiwan; Hierarchical Green-Energy Materials (Hi-GEM) Research Center, National Cheng Kung University Tainan Taiwan; Core Facility Center, National Cheng Kung University Tainan Taiwan vokhuongdien@gmail.com kilin@mail.ncku.edu.tw

## Abstract

Up to now, many guest atoms/molecules/ions have been successfully synthesized into graphite to form various compounds. For example, alkali-atom graphite intercalation compounds are verified to reveal stage-n structures, including LiC_6n_ and LiM_8n_ [M = K, Rb, and Cs; *n* = 1, 2, 3; 4]. On the other side, AlCl_4_^−^-ion/AlCl_4_-molecule compounds are found to show stage-4 and stage-3 structures at room and lower temperatures, respectively. Stage-1 and stage-2 configurations, with the higher intercalant concentrations, cannot be synthesized in experimental laboratories. This might arise from the fact that it is quite difficult to build periodical arrangements along the longitudinal *z* and transverse directions simultaneously for large ions or molecules. Our work is mainly focused on stage-1 and stage-2 systems in terms of geometric and electronic properties. The critical features, being associated with the atom-dominated energy spectra and wave functions within the specific energy ranges, the active multi-orbital hybridization in distinct chemical bonds, and atom- & orbital-decomposed van Hove singularities, will be thoroughly clarified by the delicate simulations and analyses.

## Abstract 2

Up to now, many guest atoms/molecules/ions have been successfully synthesized into graphite to form various compounds.

## Graphical abstract

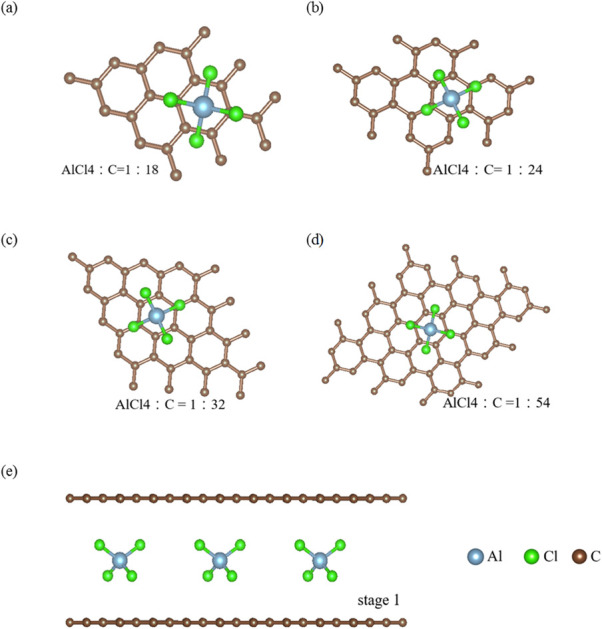

## Introduction

A pristine graphite and its intercalation/de-intercalation compounds with guest atoms/molecules/ions^1^ display rich geometric symmetries, mainly owing to chemical modifications.^2^ Bernal graphite consists of a periodical carbon-honeycomb lattice along the *z*-direction through AB stacking, *i.e.*, it is a so-called AB-stacked bulk graphite. Each graphitic sheet remains a planar structure, clearly illustrating the orthogonal features of the significant π and σ bondings. The former, which is due to the carbon-2p_*z*_ orbital hybridizations, can survive in the intralayer^3^ and interlayer [van der Waals;^4^] atomic interactions. It is responsible for the low-energy electronic properties of a 3D graphite [Fig. 2(b);^5^]. However, the strength of the latter remains the same even in the presence of interlayer couplings. As a result, it is easy to distinguish the σ and π electronic states. In general, this phenomenon keeps unchanged during the chemical reaction processes, *i.e.*, planar graphene layers exist under the very strong sp^2^–σ bondings.

**Fig. 1:**
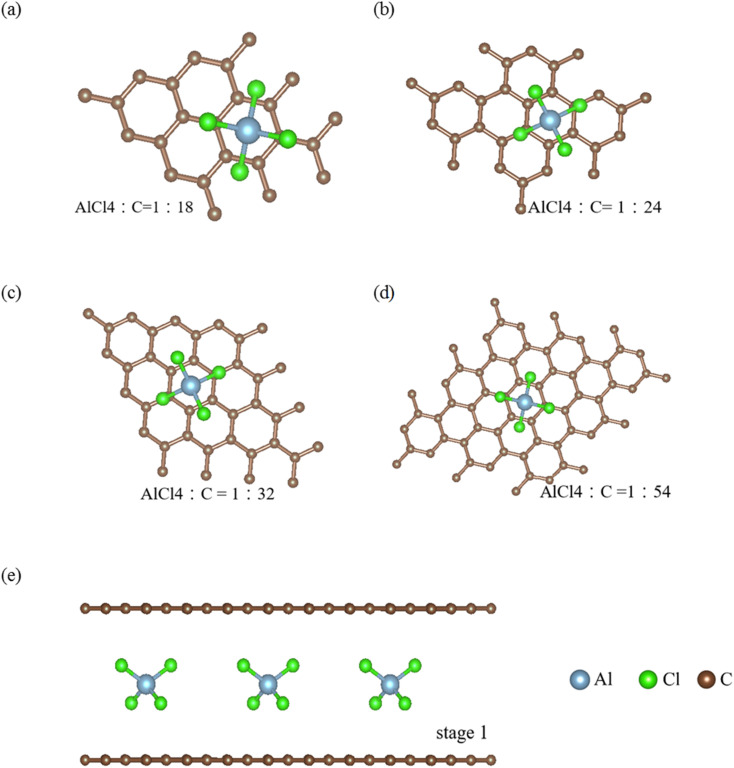
The optimal geometric structures of AlCl_4_^−^/AlCl_4_ graphite intercalation compounds with the various concentrations: (a) 1 : 18, (b) 1 : 24, (c) 1 : 32 and (d) 1 : 54 for the ration of AlCl_4_^−^/AlCl_4_ and C, and (e) the side views are shown [the light blue, light green, and dark brown ball stand for Al, Cl, and C atoms, respectively].

**Fig. 2:**
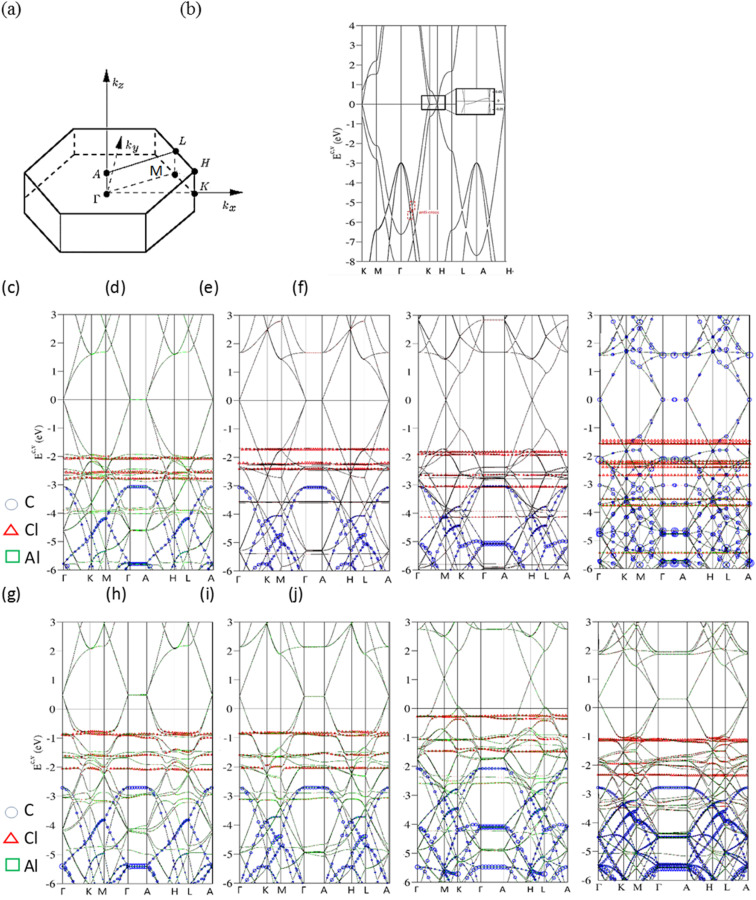
Band structures of AlCl_4_^−^-/AlCl_4_-related graphite intercalation compounds (a) along the high-symmetry points within the first Brillouin zone under the various cases: (b) a pristine system, (c)/(g) 1 : 18, (d)/(h) 1 : 24, (e)/(i) 1 : 32 and (f)/(j) 1 : 54.

**Fig. 3:**
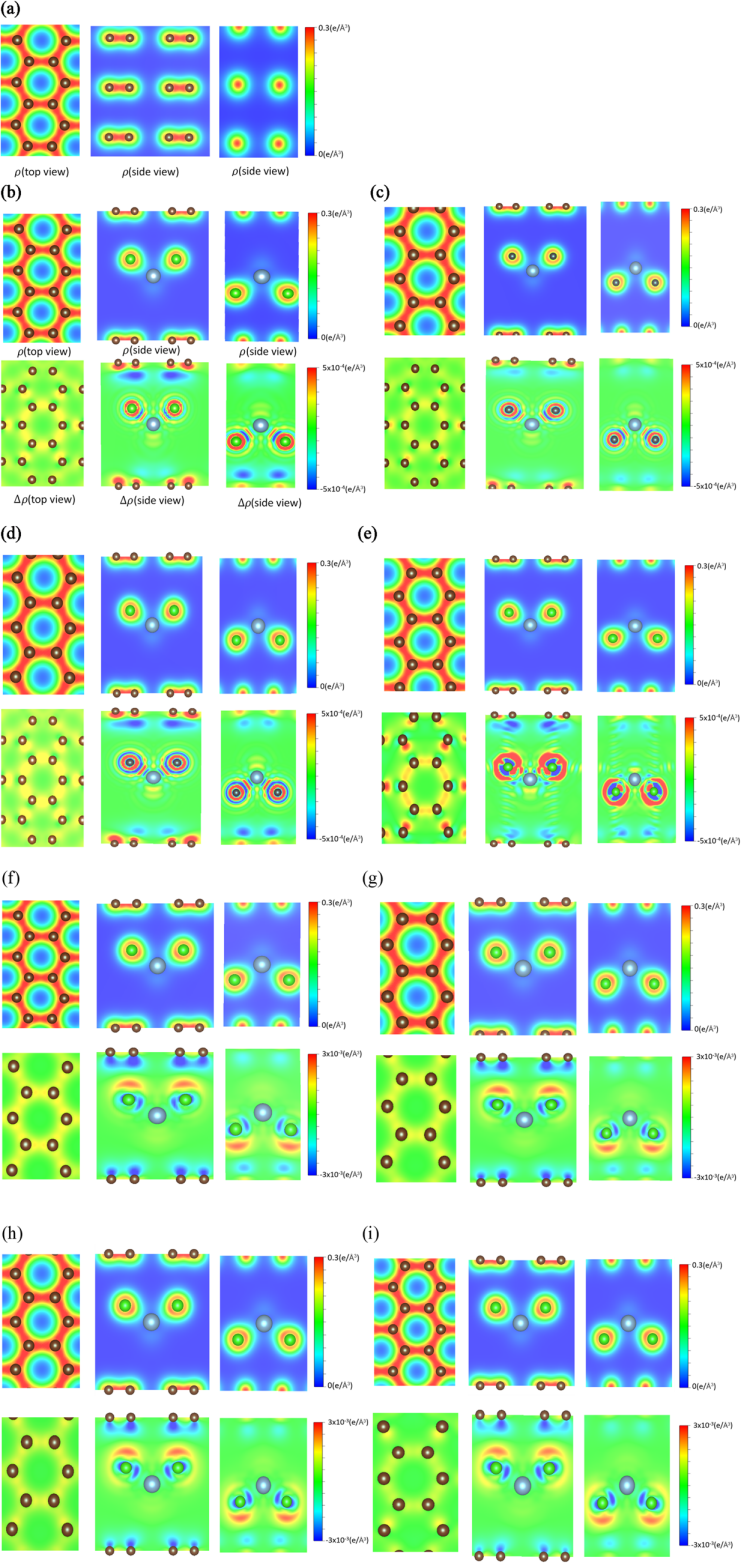
The spatial charge density distributions and their changes after AlCl_4_^−^/AlCl_4_-ion intercalation into graphite under the distinct chemical cases: (a) a pristine system, (b)/(f) 1 : 18, (c)/(g) 1 : 24, (d)/(h) 1 : 32 and (e)/(i) 1 : 54, with the top- and side views [the (*x*, *y*)-, (*x*, *z*)- and (*y*, *z*)-plane projections].

**Fig. 4:**
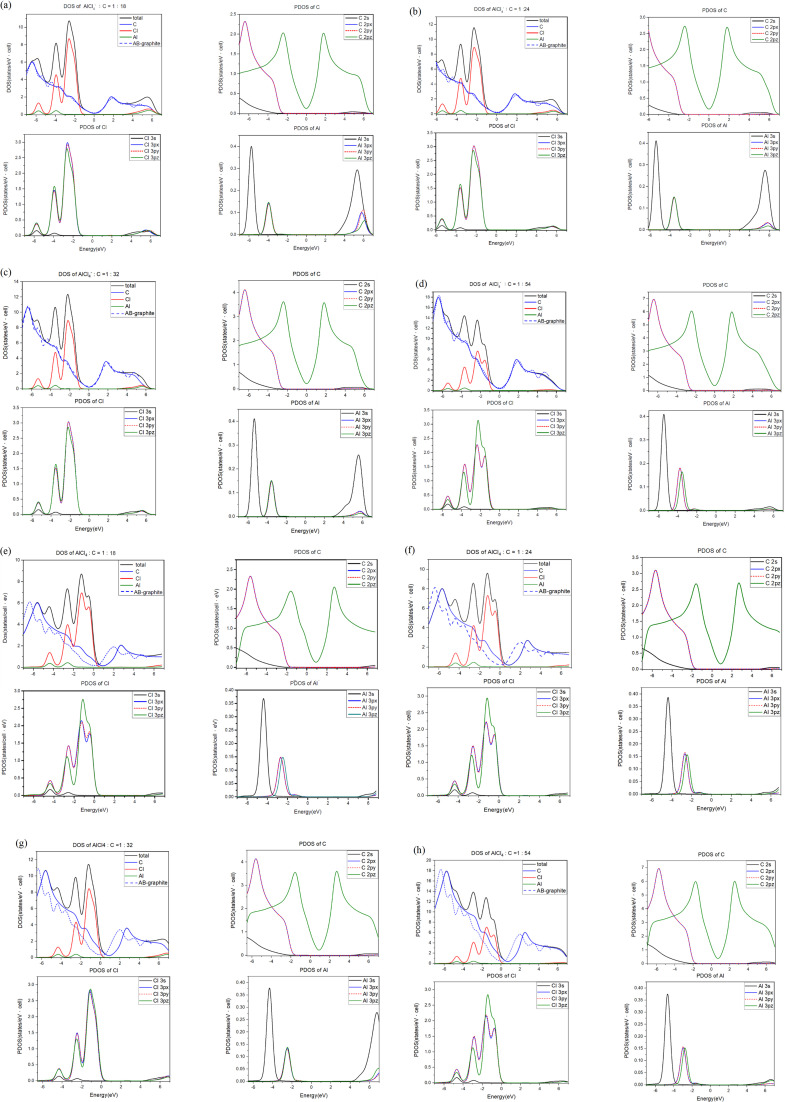
The atom- & orbital-projected density of states for AlCl_4_−/AlCl_4_-intercalation graphite compounds under different molecular concentrations: (a)/(e) 1 : 18, (b)/(f) 1 : 24, (c)/(g) 1 : 32 and (d)/(h) 1 : 54.

Apparently, the sufficiently wide spacing of ∼3.35 Å between two neighboring graphene layers, which are created by the weak, but significant van der Waals interactions [the interlayer 2p_*z*_ orbital hybridizations;^6,7^], are available for the easy intercalations of the guest AlCl^−^_4_ ions and AlCl_4_ molecules intercalations. The interlayer distance [*I*_c_ = 3.35 Å in the pristine case;^8,9^] is greatly enhanced under the various chemical environments, as clearly indicated in Tables 1 and 2 for ion and molecule intercalations, respectively. It is very sensitive the change of intercalation concentration and arrangement [Fig. 1]. As for the ion/molecule cases, *I*_c_'s are, respectively, 11.30, 11.29, 10.81 and 10.65 Å/8.77, 8.78, 8.81 and 8.80 Å for 1 : 18, 1 : 24, 1 : 36 and 1 : 54 concentrations in terms of the ratio of AlCl^−^_4_/AlCl_4_ and carbon. A very large *I*_c_ clearly indicates the thorough disappearance of the interlayer 2p_*z*_ orbital couplings. Very interestingly, the multi-orbital hybridizations in the C–AlCl^−^_4_ or C–AlCl_4_ bonds can account for the interlayer atomic interactions. As a result, there exist intralayer C–C bonds, intra-ion/intra-molecule bondings of AlCl^−^_4_/AlCl_4_, inter-ion/inter-molecule ones, and carbon-intercalant interactions. The active orbital hybridizations in distinct chemical bondings need to be identified from the other physical quantities [Fig. 2–4;^10^]. In addition, whether the van der Waals interactions can survive in large-*I*_c_ graphite intercalation compounds requires a very detailed numerical examination.

**Table 1:** The optimal geometric structures of AlCl_4_^−^-graphite intercalation compounds with the various concentrations: (a) 1 : 18, (b) 1 : 24, (c) 1 : 32 and (d) 1 : 54 for the ration of AlCl_4_^−^ and C

	Concentration (AlCl_4_/C)	Layer-distance (Å)	C–C bond-length (Å)	Al–Cl bond-length (Å)	Al–Cl bond-angle (°)
Primitive		3.35	1.42	2.159	109.0
1 : 54	1.85%	10.65	1.424	2.165	108.01
1 : 32	3.12%	10.81	1.426	2.163	107.94
1 : 24	4.16%	11.29	1.427	2.162	107.46
1 : 18	5.55%	11.30	1.428	2.160	107.19

**Table 2:** The similar results in Table 1, but illustrated for AlCl_4_-graphite intercalation compounds. The blue shifts of the Fermi levels under the various concentrations are also shown for the charge transfer effects

	Concentration (AlCl_4_/C)	Layer distance(Å)	C–C bond-length (Å)	Al–Cl bond-length (Å)	Al–Cl bond-angel (°)	Blue shifts (eV)
Primitive		3.35	1.42	2.159	109.27	
1 : 54	1.85%	8.80	1.421	2.165	113.03	0.978
1 : 32	3.12%	8.81	1.422	2.163	112.00	0.911
1 : 24	4.16%	8.78	1.421	2.162	111.45	0.862
1 : 18	5.55%	8.77	1.422	2.160	111.7	0.753

The regular arrangements of large ions/molecules, which possess high projection symmetries, are chosen for a model study. Very interestingly, a pristine graphite has a periodical AB stacking configuration along the *z*-direction, being dramatically transformed into an AA one during the strong chemical intercalations/de-intercalations. This will lead to drastic changes of the other fundamental properties.^11^ As for each intercalant, an aluminum atom is just situated at the hollow site above a hexagon [the top view of the *x*–*y* plane projection in Fig. 1]; furthermore, it is accompanied by two chloride atoms along the dimer and bridge-middle directions simultaneously. The periodical ion/molecule distribution fully occupies the whole interlayer spacing so that the intercalant layer is formed after the chemical modification.^12,13^ This is the so-called stage-1 graphite intercalation compound, where there is only monolayer graphene between two neighboring intercalant layers. And then, the intercalant concentrations decline within the larger unit cells. There are larger Moire superlattices in the dilute cases.^14^ These graphite de-intercalation systems are expected to have lower lattice symmetries, and thus more complicated calculations/phenomena^15^ compared with alkali atoms.^16^ Most importantly, the atomic configurations in the saturated ions and the unbalanced molecules [the excited angling bonds]^17^ play a critical role in the obvious difference in the interlayer distances [∼11 Å in Table 1 and ∼8.8 Å in Table 2], since the carbon-intercalant interactions are weaker under the former chemical environment. During the concentration variation of large ions/molecules, the sensitive dependences of their internal bonding angles indicate the significant contributions to the total ground state energies. Specifically, the ion–ion interaction might strongly modify the optimal interlayer distances.

The intercalant configurations deserve a closer examination. Previous experimental^18^ and theoretical^19^ studies have proposed the characterizations of stage-*n* systems during the chemical modification processes, mainly owing to the unchanged σ bonding honeycomb lattices^20^ and the drastic changes of free carrier densities.^21^ As for *n* ≥ 2 cases, intercalants exhibit a periodical distribution along the *z*-direction, but a non-uniform one. However, this unusual configuration might not agree with the natural ion/molecule diffusion phenomena under external factors [*e.g.*, pressure and thermal energy; ^22^]. It is mission impossible to create the critical mechanisms in forbidding their intercalations and de-intercalations inside any spacings of the nearest-neighbor graphitic layers. That is, the guest-intercalant transport, which obeys the thermal dynamical laws,^23^ will be revealed in the successfully synthesized compounds. Obviously, there are certain important differences between stage-*n* graphite intercalation compounds and stage-1 systems^24,25^ with various concentrations, covering the active chemical bonds, the interlayer distances, the crystal symmetries of the Moire superlattices, the bonding angles, the charge transfers, and the metallic or semiconducting behavior. The problem is how to clarify which kind of stacking configuration is the optimal one after the experimental synthesis. This interesting issue can be settled through the method of molecular dynamics,^26^ in which the physical/chemical/materials environments are necessary conditions for delicate numerical simulations. Systematic investigations are required in near-future basic science research.

Very apparently, high-precision X-ray diffraction spectroscopy, as clearly illustrated in Chap. 3, is reliable in fully exploring the optimal crystal structures of AlCl^−^_4_/AlCl_4_ graphite intercalation compounds. The examined quantities cover the periodical distances along the *z*-direction and the lattice constants on the (*x*, *y*) plane. Whether this method can detect the intercalant-dependent bonding angles is worthy of further thorough investigations. The up-to-date X-ray patterns claim the successful observations of stage-3 and stage-4 large-intercalant graphite intercalation compounds.^18^ The former/the latter is deduced to be relatively stable at lower/room temperatures [∼250 K/300 K] during the charging and discharging processes in aluminum-ion-based batteries.^18^ Apparently, temperature is one of the critical factors in determining the lattice symmetries. For example, the thermal excitation energies are expected to be comparable to the interlayer graphene–intercalant interactions. The theoretical predictions on stage-1 systems with various intercalant concentrations can be generalized for stage-*n* ones. The stacking configurations would strongly modify the similar physical and chemical phenomena [great enhancement or reduction of the similar quantities]. This is under a current investigation.^27^

## Theoretical calculations

The first-principles simulations within DFT by solving the Kohn–Sham equations are dominating methods for the investigation of the fundamental properties of periodic systems, *i.e.*, they are frequently utilized to study the geometric, electronic, magnetic, and optical properties. The Perdew–Burke–Ernzerh formula is utilized to deal with the many-particle Coulomb effects. The first Brillouin zone is sampled by 9 × 9 × 9 and 100 × 100 × 100 *k*-point meshes within the Monkhorst–Pack scheme, respectively, for the optimal geometry and band structure. Moreover, the convergence condition of the ground state energy is set to be ∼10^−5^ eV between two consecutive evaluation steps, where the maximum Hellmann–Feynman force for each ion is below 0.01 eV Å^−1^ during the atom relaxations. We also take the van der Waals dispersion correction into consideration with the set IVDW = 1 in INCAR, which is performed on the DFT-D2 method of Grimme.

## Results and discussions

### Rich and unique electronic properties

Electronic properties of AlCl_4_^−^-/AlCl_4_-graphite intercalation compounds, 3D band structures, charge density distributions and density of states, are fully explored by the numerical VASP calculations and further generalized by the phenomenological models. The critical mechanisms, the intralayer π- and σ-bondings, the interlayer van der Waals interactions, the carbon-intercalant orbital hybridizations, and the intra- and inter-ion/inter-molecule interactions, are examined and identified by the delicate analyses. These will be revealed in semi-metallic or metallic behavior [the density of free carriers due to the weak or strong charge transfer], the well-characterized/unusual π or σ bands, the C-, Al- and Cl-dominated energy spectra at different energy ranges, the spatial orbital bondings after and before intercalations, and the merged special structures of the energy-dependent van Hove singularities.

Bernal graphite and AlCl_4_^−^, AlCl_4_ graphite intercalation compounds, as clearly shown in Fig. 2, present diverse electronic energy spectra and wave functions. Their first Brilloun zone in Fig. 2(a) possesses a hexagonal symmetry,^28^ in which the (*kx*, *ky*)-projection is similar to that of a layered graphene.^29^ The band structure of a pristine graphitic system [Fig. 2(b)], being illustrated along the high-symmetry points [*ΓKMΓAHLA*; ^30^], is rich and unique. This system is an unusual semimetal, while a monolayer graphene is a zero-gap semiconductor with a vanishing density of states at the Fermi level.^31^ This difference obviously indicates the important role of the interlayer van der Waals interactions. The intralayer and interlayer C-2p_*z*_ orbital hybridizations, respectively, create the gapless Dirac-cone structure^32^ and the weak but significant overlaps of the valence and conduction bands.^33^ The latter are responsible for the asymmetric energy spectra of valence holes and conduction electrons about the Fermi level. The low-lying π electronic states are initiated from the *K* and *H* valleys [the inset of Fig. 2]. Furthermore, their state energies are, respectively, lower and higher than the Fermi energy in terms of a weak energy dispersion, *i.e.*, there exist 3D free carriers of the hole and electron pockets. Very interestingly, the other essential properties are easily further modulated by external factors, such as the intercalation-/de-intercalation-,^34^ temperature-, pressure-, and magnetic-field-enriched phenomena.^35–37^ In addition, the systematic investigations on bulk graphite systems can be found in review articles and books.^38^ Most importantly, the well-behaved π bands can be clearly identified in the energy spectra along *KMΓ* and *HLA* with the whole widths more than 7 eV. The wide π-band widths are attributed to a close cooperation of the intralayer and interlayer carbon-2p_*z*_ orbital hybridizations. On the other side, the *σ* orbitals of [2p_*x*_, 2p_*y*_, 2s] could build two degenerate bands and one band below at the *Γ* point below −3 and −10 eVs, respectively. These features are mainly determined by the σ-electronic hopping integrals and ionization energies.^39^

AlCl_4_^−^-ion and AlCl_4_-molecule intercalations, which are, respectively, shown in Fig. 2(c)–(j), are able to create the diverse energy spectra and wave functions. The dramatic changes cover the variation of high-symmetry points, the creation of a lot of energy subbands, the greatly enhanced asymmetry of occupied and unoccupied spectra about the Fermi level, the obvious reduction or enhancement of band overlaps [the diversified free carrier densities], the almost isotropic/highly anisotropic features near/away from *E*_F_ = 0, the various energy dispersions with the different critical points, the frequently crossing and anti-crossing behaviors, the non-well-behaved π–σ-band widths, the carbon-, aluminum- and chloride-dominances at the different energy ranges [blue circles, red triangles and green squares, respectively]. Moreover, the π and σ electronic states can be easily identified from the original valleys, but not their whole band widths. After the chemical modifications, the enlarged Moire superlattices possess many atoms/ions in primitive unit cells [Fig. 1], so that the hexagonal first Brillouin zone in Fig. 2(a) is diminished quickly, especially for the low concentration cases. This leads to many valence and conduction bands with smaller wave-vector ranges.^40^ Due to zone-folding effects,^41^ the low-lying electronic states are initiated from the *Γ* and *A* valleys [the *K* and *H* ones] under the cases of 1 : 18, 1 : 24 and 1 : 54 [1 : 32]. As to the ionic chemical environments, a pair of anisotropic valence and conduction bands across the Fermi level, which appears in a pristine Bernal graphite [Fig. 2(b)], is changed into the isotropic Dirac-cone structure of monolayer graphene. Furthermore, the energy spectra are dispersionless along the *ΓA* or *KH* directions. This clearly illustrates the semiconducting behavior with a zero-band gap and density of state at *E*_F_ = 0, simultaneously indicating the very weak carbon-intercalant-ion orbital hybridizations under the saturated atomic configurations. These graphite intercalation compounds are expected to present the lower electrical conductivities after the chemical reactions,^42^ However, they become outstanding merits in aluminum-ion transports and cathode intercalations/de-intercalations,^43^ Very interestingly, the π-electronic dominance in the energy range of *E*^c,v^ ≤ 1.0 eV also comes to exist in the AlCl_4_-molecule graphite intercalations, the obvious red shifts of *E*_F_'s is revealed in any chemical cases. Electrons are largely transferred from carbon atoms to molecules, where the latter possess the larger affinities.^44^ The strong p-type doping effects should be attributed to the significant carbon-molecule orbital hybridizations. In short, three types of band structures, semimetal, semiconductor and metal, respectively, arise from the interlayer van der Waals, carbon-saturated-ion and carbon–molecule interactions. Whether similar phenomena can be found in other graphite intercalation compounds deserves a closer VASP simulation.

The atom dominance, which corresponds to the spatial distribution probability of each wave-vector state, is clearly revealed in the specific energy ranges. It is determined by the intrinsic orbital hybridizations of chemical bonds. Most of the electronic states in the entire energy spectrum is dominated by carbon atoms for the various AlCl_4_^−^-ion and AlCl_4_-molecule intercalations [blue open circles in Fig. 2(c)–(j) for the π, σ and π* energy subbands]. This is attributed to the dominating π, σ, carbon-intercalant bondings, since their initial state energies remain there and the frequent anti-crossing behavior comes to exist.^45^ Specifically, aluminum atoms make observable contributions near *E*^v^ ∼ −4 and −6 eVs [green squares], suggesting the linking roles through a large ion/molecule structure. As for chloride atoms, the unusual roles are revealed as the weakly dispersive valence bands, at least four bands, below the Fermi level more than 1 eV [red triangles]. Their main features, energy, degeneracy, spacing and group velocity of the valence subband, are very sensitive to the change of intercalant configuration. The partially flat subbands, with the zero velocities [localized behavior] frequently appear under the large-ion intercalations [Fig. 2(c)–(f)]. However, they might exhibit observable modifications in the molecular cases [Fig. 2(g)–(j)]. The larger carrier mobility clearly indicates the more extensive charge distributions. The above-mentioned characteristics might be closely related to all the active chemical bondings, being supported by the further discussions of the charge density distributions [Fig. 3] and van Hove singularities [Fig. 4].

The theoretical predictions of occupied electronic states below the Fermi level can be examined by angle resolved photoemission spectroscopy [ARPES;^46^], as discussed in Chap. 3.3 in detail. In general, it is very difficult to measure the *k*_*z*_ – dependent energy spectra because of the destruction of the momentum of conservation through the surface boundary How to utilize the most important band features along *KH*, *ML* and *ΓA* would become a critical technique of identifying the energy dispersions. The high-resolution ARPES measurements have been successfully conducted on the semi-metallic energy bands in Bernal graphite, but not those of the rhombohedral and simple hexagonal graphites [ABC- and AA-stacked ones; ^47,48^]. According to the calculated results, the second and third systems, respectively, the lowest and highest free carrier densities, are mainly the result of the symmetry of the stacking configuration.^49^ As to the AlCl_4_^−^-ion/AlCl_4_-molecule intercalation of stage-3/stage-4 graphite intercalation compounds, the observed occupied energy spectra are expected to exhibit greatly diversified phenomena in terms of stacking-symmetry dependences, red shifts of the Fermi level, band overlaps [free carrier densities], strong energy dispersions, high anisotropies, and the characterizations of π- and σ-electronic energy spectra. In addition to these features, VASP simulations on the stage-1 systems are able to provide very useful information about the chloride- and aluminum-related valence bands. Further experimental examinations are very helpful to thoroughly clarify the intercalation/de-intercalation effects on electronic energy spectra and wave functions, as well as the intrinsic quasiparticle properties of orbital hybridizations.^50^

### The active orbitals hybridizations

The spatial charge distributions [*ρ*(*r*)*s*] and their variations [Δ*ρ*(*r*)*s*] after the chemical intercalations, as clearly shown in Fig. 3, provide very useful chemical pictures for fully comprehending the critical chemical bonds of the active orbital hybridizations. The [*x*, *y*]-top, [*x*, *z*]-side and [*y*, *z*]-side views fully illustrate the rich and unique intrinsic interactions: the C–C bonds in a honeycomb lattice, the interlayer C–Cl bonds, and the intra-ion/intra-molecule Al–Cl and Cl–Cl bonds. The significant chemical bondings agree with the density of states [the merged van Hove singularities in Fig. 4] and band structures [atom dominances in Fig. 2]. The strong evidence is thoroughly identified from *ρ*(*r*)*s* and Δ*ρ*(*r*)s. First, the prominent σ bonding, which survives in pristine Bernal graphite is revealed as a very strong covalent bond between two neighboring carbon atoms [a super-high charge density as indicated by the red color on the [*x*, *y*] plane in Fig. 3(a); ^51^]. Furthermore, the π bonding is characterized by the wave-like charge distribution *ρ*(*r*)*s* and their variations Δ*ρ*(*r*)s due to the significant parallel 2p_*z*_-orbital hybridizations, as examined from *ρ*(*r*) on both the [*x*, *z*] and [*y*, *z*] planes [Fig. 3(a)]. Its distribution along the *z*-direction is somewhat extended by the interlayer van der Waals interactions.^52^ Secondly, the significant carbon-intercalant couplings, corresponding to the large-ion cases [Fig. 3(b)–(e)], are directly reflected in the strongly anisotropic charge distributions *ρ*(*r*)*s* in the 1st, 2nd and 3rd plots] and their variations Δ*ρ*(*r*)*s* near the chloride atoms and the drastic changes between them, especially for the [*x*, *z*]- and [*y*, *z*]-plane projections. On the other hand, the large-molecule intercalations, as indicated in Fig. 3(f)–(i), can greatly enhance the charge density distribution in C–Cl bonds, since its atomic configuration belongs to the non-closed-shell states. As for the Al–Cl and Cl–Cl bonds, they present prominent bondings through the obvious distorted charge densities near chloride and aluminum atoms. In addition, it is very difficult to examine the existence of AL–C and Al–Al bonds from the results. Furthermore, the calculations cannot provide enough information for examining the effects of the inter-ion/inter-molecule intercations. The similar analyses can be generalized for other multi-component graphite intercalation compounds, *e.g.*, the active chemical bonds in H_2_SO_4_^−^,^53^ HNO_3_^−^,^54^ and FeCl_3_^−^^55^ related systems.

According to the well-defined density of states, *D*(*E*) is expressed as the integration of the inverses of group velocities on the constant-energy configuration. For example, the 3D/2D/1D are greatly enriched by the various first derivatives of the gradient operations on electronic energy spectra and the specific integrations on the closed shells/circles/two discrete wave-vector points. A vanishing group velocity comes to exist and corresponds to a critical point in the energy-wave-vector space. The singular integration function leads to a special structure, namely, a van Hove singularity. The main features of singular structures, *i.e.*, their forms, intensities, energies and numbers, are very sensitive to the characteristics of the distinct critical points and dimensionalities.^56^ In general, the former are classified into the extreme, saddle and partially flat points, being clearly illustrated by the linear, parabolic, almost dispersionless and sombrero-shape energy dispersions of few-layer graphene systems.^57^ When the orbital- and orbital-decomposed density of states are calculated for any condensed-matter systems, the various singular structures, with prominent intensities, are available for determining the active orbital hybridizations of different chemical bonds.^58^ This is based on their great enhancements through the emerged van Hove singularities.^59^ As for large-intercalant graphite intercalation compounds, there are a plenty of atom- and orbital-projected components. The very complicated results need to be analyzed in detail.^60^

For each intercalation case of AlCl_4_^−^/AlCl_4_, there are one atom- and three orbital-decomposed density of states, being rather sufficient in providing useful information about the active multi-/single-orbital hybridizations of the distinct chemical bonds.^61^ Very apparently, Fig. 4 shows the rich and unique van Hove singularities mainly due to [C, Cl, Al] atoms and their significant orbitals. The magnitude of *D*(*E*) at the Fermi level represents the characteristics of free carriers. Bernal graphite and ion intercalation systems have low values at *E*_F_ = 0, as well as band structures, respectively, suggesting the semi-metallic and semiconducting behaviors. However, each large-molecule case exhibits a finite value there. Most importantly, the difference between the Fermi level and the featured energy with the smallest density of states could be regarded as its red shift [details in Table 2]. Furthermore, this covered area just corresponds to the total free carriers per unit cell after the obvious charge transfers from carbon to chloride [Fig. 3]. The stronger affinity of the latter is responsible for the p-type doping effects [free valence holes;^62^]. This quantity is deduced to be proportional to the AlCl_4_-molecule concentration. In addition, the Fermi-momentum states of electronic spectra [Fig. 2(g)–(j)] are not reliable in evaluating the transferred valence hole density in the presence of complicated zone-folding effects.

The significant chemical bonds and their active multi-/single-orbital hybridizations are further achieved from the delicate analyses, covering all the separated and merged van Hove singularities [Fig. 4]. The concise physical and chemical pictures are also supported by the electronic energy spectra [Fig. 2] and the spatial charge distributions [Fig. 3]. Both AlCl^−^_4_ and AlCl_4_ graphite intercalation compounds possess intralayer carbon–carbon, interlayer carbon-intercalant, and intra-/inter-intercalant interactions, respectively, leading to the C–C, C–Cl, Al–Cl and Cl–Cl bonds. However, observable evidence of the merged van Hove singularities is absent for Al–C and Al–Al. The prominent chemical bondings are thoroughly illustrated as follows. Since AlCl^−^_4_ has a closed 0 shell atomic configuration, each graphitic sheet recovers to a pure honeycomb lattice. The π- and σ-electronic spectra are well separated from each other [all PDOS of C cases in Fig. 4]. Furthermore, the former [the pink curves] and the latter [the red, blue and black curves] are, respectively, characterized by the initial/prominent structures at ∼−2.0 eV and ∼−3.12/−6.15 eV. In addition, two strong peaks mainly arise from the saddle points of the valence π and σ bands. Apparently, π and sp^2^ bondings are orthogonal to each other and thus survive in the C–C bonds. The interlayer C–Cl bonds are revealed as the multi-orbital hybridizations of [2p_*x*_, 2p_*y*_, 2p_*z*_]–[3p_*x*_, 3p_*y*_, 3p_*z*_] through the emerged structures within −6.1 eV ≤ *E* ≤ −1.8 eV. As for the Al–Cl/Cl–Cl bonds, the obvious four-orbital hybridizations of [3s, 3p_*x*_, 3p_*y*_, 3p_*z*_]−[3s, 3p_*x*_, 3p_*y*_, 3p_*z*_]/[3s, 3p_*x*_, 3p_*y*_, 3p_*z*_]–[2s, 2p_*x*_, 2p_*y*_, 2p_*z*_] are clarified from the van Hove singularities at −4.0 eV and −5.9 eV. Such unusual results are due to the fact that 3s and [3p_*x*_,3p_*y*_,3p_*z*_]-decomposed, respectively, appear at the same and different energies for Al and Cl. The similar features of the density of states can be found in the molecular intercalation cases, while the red-shift phenomena are created by the very strong p-type doping effects. That is, the important C–Cl bonds can enrich the valence van Hove singularities near the Fermi level. This is consistent with more charge variations between the honeycomb lattice and the intercalant layer. In addition, it is almost impossible to investigate the inter-ion and inter-molecule interactions from the van Hove singularities.^63^

## Conclusions

In summary, Bernal graphite, stage-1 AlCl_4_^−^-ion and AlCl_4_-molecule graphite intercalation compounds exhibit diverse quasiparticle behavior under the distinct orbital hybridizations of intralayer and interlayer chemical bonds. The effects, which are due to significant van der Waals interactions, rich intercalations of closed-shell ions, and strong charge transfers, are responsible for the rich and unique properties. They are directly reflected in the distinct crystal symmetries [the periodical configurations perpendicular/on the (*x*, *y*) plane], the largely enhanced Moire superlattices, the existence of many valence and conduction energy subbands [zone-folding effect], the variations about the initial high-symmetry valleys, the largely enhanced asymmetry of the hole and electron spectra about the Fermi level, the vanishing band overlaps/the p-type doping effect [the zero/finite free carrier densities after ion/molecule interactions], the almost isotropic/highly anisotropic features close/away from the Fermi level, the various energy dependences at the distinct critical points, the frequent band crossings and mixings [the complicated orbital hybridizations], the non-well-defined π- and σ-electronic band widths, and the carbon-, aluminum- and chloride-determined energy spectra at the distinct energy ranges. The active multi-/single-orbital hybridizations of C–C/C–Cl/Al–Cl/Cl–Cl chemical bonds are identified to be [2s, 2p_*x*_, 2p_*y*_]–[2s, 2p_*x*_, 2p_*y*_] & 2p_*z*_−2p_*z*_/[2s, 2p_*x*_, 2p_*y*_, 2p_*z*_]−[3s, 3p_*x*_, 3p_*y*_, 3p_*z*_]/[3s, 3p_*x*_, 3p_*y*_, 3p_*z*_]–[3s, 3p_*x*_, 3p_*y*_, 3p_*z*_]/[3s, 3p_*x*_, 3p_*y*_, 3p_*z*_]–[3s, 3p_*x*_, 3p_*y*_, 3p_*z*_]. However, observable evidence is very difficult to examine for the C–Al and Al–Al bonds. Up-to-date experiments only verify the observation of stage-3 and stage-4 cases,^64,65^ in which the molecular dynamics could be used to examine the existence of stage-1 and stage-2 configurations. Concerning the complicated intercalation and de-intercalation processes, their critical roles on the large-ion transports within high-performance batteries require for the systematic investigations,^66^ especially with regard to the development of a theoretical frameworks.

## Conflicts of interest

There are no conflicts to declare.

## Supplementary Material
